# Current Research Progress of the Role of LncRNA LEF1-AS1 in a Variety of Tumors

**DOI:** 10.3389/fcell.2021.750084

**Published:** 2021-12-20

**Authors:** Qingyuan Zheng, Xiao Yu, Menggang Zhang, Shuijun Zhang, Wenzhi Guo, Yuting He

**Affiliations:** ^1^ Department of Hepatobiliary and Pancreatic Surgery, The First Affiliated Hospital of Zhengzhou University, Zhengzhou, China; ^2^ Key Laboratory of Hepatobiliary and Pancreatic Surgery and Digestive Organ Transplantation of Henan Province, The First Affiliated Hospital of Zhengzhou University, Zhengzhou, China; ^3^ Open and Key Laboratory of Hepatobiliary and Pancreatic Surgery and Digestive Organ Transplantation at Henan Universities, Zhengzhou, China; ^4^ Henan Key Laboratory of Digestive Organ Transplantation, Zhengzhou, China

**Keywords:** long non-coding RNA, LEF1-AS1, cancer biomarker, function, molecular mechanism

## Abstract

Long non-coding RNAs (lncRNA), as key regulators of cell proliferation and death, are involved in the regulation of various processes in the nucleus and cytoplasm, involving biological developmental processes in the fields of immunology, neurobiology, cancer, and stress. There is great scientific interest in exploring the relationship between lncRNA and tumors. Many researches revealed that lymph enhancer-binding factor 1-antisense RNA 1 (LEF1-AS1), a recently discovered lncRNA, is downregulated in myeloid malignancy, acting mainly as a tumor suppressor, while it is highly expressed and carcinogenic in glioblastoma (GBM), lung cancer, hepatocellular carcinoma (HCC), osteosarcoma, colorectal cancer (CRC), oral squamous cell carcinoma (OSCC), prostatic carcinoma, retinoblastoma, and other malignant tumors. Furthermore, abnormal LEF1-AS1 expression was associated with tumorigenesis, development, survival, and prognosis via the regulation of target genes and signaling pathways. This review summarizes the existing data on the expression, functions, underlying mechanism, relevant signaling pathways, and clinical significance of LEF1-AS1 in cancer. It is concluded that LEF1-AS1 can serve as a novel biomarker for the diagnosis and prognosis of various tumors, thus deserves further attention in the future.

## Introduction

Cancer is a major global public health problem (Siegel et al., 2020; Li N. et al., 2021; Leyva-González et al., 2021), which has become the leading cause of death in China (Li T. et al., 2020). Recently, the morbidity and mortality of cancer have been clearly on the rise in the country, comprising a significant health burden that it is likely to further increase (Islami et al., 2017). Altered gene expression is a major feature of many cancers, thus it has been extensively studied. RNAs are essential for gene expression (Yin et al., 2017), whether they are protein-encoded RNAs (mRNAs) or non-encoded RNAs involved in the regulation of transcription, such as long noncoding RNAs (lncRNAs) (Goodall and Wickramasinghe, 2021). In recent years, there has been a great deal of research into the role of lncRNAs in malignant tumors (Li G. et al., 2021; Guo et al., 2021; Hou and Peng, 2021; Teng et al., 2021), which proved that lncRNAs are key regulators of cancer-associated pathways and thus are important disease biomarkers (Liu S. J. et al., 2021).

Long-chain non-coding RNAs are a group of RNA molecules, which are over 200 nucleotides long and lack the protein coding function (Silva et al., 2019; Wang P.-S. et al., 2021). They are involved in a series of processes that regulate tumor biology, and play important roles in regulating oncogenes or tumor suppressor genes (Huang et al., 2017). Researchers have reported the abnormal expression of lncRNAs in a variety of cancers, indicating their important role in regulating cancer cell proliferation, chemotherapy resistance, and metastasis. For example, LINC00504 is upregulated and promotes tumor cell proliferation and migration in breast cancer (Hou et al., 2021). LncRNAs are often used as competitive endogenous RNA (ceRNA) to regulate gene expression by specifically sponging the corresponding microRNA to establish a large regulatory system across the transcriptome (Wang X. et al., 2021; Chen et al., 2021). The regulated target genes are widely involved in multiple signaling pathways, most of which are closely related to tumors (Gao et al., 2021).

LEF1 (lymph enhancer-binding factor 1), also known as a nuclear transcription factor, is normally expressed in T cells and pro-B cells (Kühnl et al., 2011). LEF1 antisense RNA 1 (LEF1-AS1) is a recently discovered lncRNA located in the 4q25 chromosome and encoded in the lymphoid enhancer-binding factor 1 (LEF1) locus. LEF1 was shown to be highly expressed in several cancer types, such as ovarian cancer (Zhang and Ruan, 2020), lung cancer (Wang et al., 2019), and liver cancer (Gao et al., 2020), etc., and regulate tumor development and progression. Besides, LEF1 could increase the osteogenic differentiation of dental pulp stem cells through the regulation of miR-24-3p (Wu et al., 2020). LEF1-AS1 can also regulate the proliferation and migration of vascular smooth muscle cells by targeting the miR-544a/PTEN axis (Zhang L. et al., 2019). Moreover, LEF1-AS1 is a conserved transcript of hematopoietic dysfunction. Studies have indicated that it is associated with higher-risk myelodysplastic syndrome (Szikszai et al., 2020).

This review mainly discusses the biological functions of LEF1-AS1 and the related molecular mechanisms in a variety of different tumors, primarily elaborating on clinical and animal models, as well as cell experiments, and focusing on summarizing the clinical significance of LEF1-AS1, its functions in tumors, and the related signaling pathways. Several major pathways associated with LEF1-AS1 are further discussed. This paper intends to provide a reference for finding new and feasible targets for tumor treatment and assist with the search for biomarkers.

## Studies on LEF1-AS1 Using Clinical Patients, Animal Models and Cell Experiments

A large number of studies have reported that LEF1-AS1 is abnormally expressed in various human cancers, essentially manifested as increased expression, which was often associated with the poor prognosis of tumors. In addition, LEF1-AS1 was shown to play a key role in a variety of biological processes, such as proliferation (Xiang et al., 2020), invasion (Wang et al., 2019; Xiang et al., 2020), migration, angiogenesis (Li W. et al., 2020; Dong et al., 2020), and apoptosis. On this basis, we consider the research progress on the role of LEF1-AS1 in tumors from the perspectives of clinical patients, mouse models and cell experiments.

## Human Studies

Clinical studies have shown that LEF1-AS1 expression is significantly increased in a variety of malignant tumors, and is closely related to tumor size, TNM staging, histological grade, lymph node metastasis, and overall survival (OS). Most of these studies have used adjacent normal tissues as control (Supplementary Table S1).

### Glioma

Glioma is one of the most prevalent types of primary intracranial carcinoma, which includes astrocytoma, glioblastoma multiform (GBM), oligodendrogliomas, and mixed tumors (Peng et al., 2018; Zhang et al., 2021). In particular, GBM has a high degree of malignancy, rapid clinical course, poor prognosis, and a median survival time of fewer than 1.5 years. Currently, there is no effective targeted therapy for GBM (Osswald et al., 2015). Many studies have shown that abnormal gene expression is pivotal in the development of glioma (Steponaitis et al., 2016), which is accompanied by the increase of abnormal expression of functional lncRNAs. Chen et al. pointed out that the TCGA database and the comparison of cancerous and paracancerous tissues of 40 patients with glioma showed that LEF1-AS1 was significantly upregulated in cancer tissues, and this was significantly associated with the poor survival rate of GBM patients, indicating the potential malignancy roles of LEF1-AS1 in glioma (Cheng Z. et al., 2020). Other studies have also confirmed that LEF1-AS1 was abnormally expressed in GBM, and the 5-year OS period of GBM patients with high LEF1-AS1 expression was significantly shortened (*p* < 0.0001) (Wang et al., 2017).

### Lung Cancer

The leading cause of cancer-related deaths is lung cancer worldwide (Thai et al., 2021), which is classified into two major histological types: small cell lung cancer (SCLC) and non-small cell lung cancer (NSCLC) (Tan et al., 2016). LncRNAs were recently identified as the primary regulators of initiation, progression, and therapeutic response in a variety of cancers (Zhang et al., 2013). Many studies have shown that LEF1-AS1 is highly expressed in lung cancer, has oncogenic function, and may be a potential target for research and treatment (Wang et al., 2019; Yang et al., 2019; Xiang et al., 2020).

To investigate the role of LEF1-AS1 in lung cancer, the expression of LEF1-AS1 in lung cancer specimens and normal tissues adjacent to cancer was first detected by qRT-PCR. Wang et al. found that the expression level of LEF1-AS1 in tumor tissues was significantly higher than that in paracancerous tissues. Further studies have proved that the overall survival rate of patients with high LEF1-AS1 expression is much lower than that of patients with low LEF1-AS1 expression (Wang et al., 2019). Yang and his team also pointed out that the upregulation of LEF1-AS1 in NSCLC tissues affects the prognosis of patients with NSCLC; the group with high LEF1-AS1 was significantly correlated with tumor size (*p* = 0.037) and TNM stage (*p* = 0.04) (Yang et al., 2019).

### Hepatocellular Carcinoma

The most common form of liver cancer is HCC, which accounts for approximately 90% of cases (Llovet et al., 2021). According to global cancer statistics, HCC is the fifth most common cancer and the third most common cause of cancer-related deaths (Jiang et al., 2019; Thuluvath et al., 2021). From a strictly oncologic standpoint, liver transplantation is the best treatment for HCC that is confined to the liver. However, this approach is unsuitable for a wide range of HCC patients due to its high cost and limited ligands (Vibert et al., 2020). Therefore, further studies are urgently needed to determine the best therapeutic regimen for HCC. LncRNAs have been recognized as cancer-related biomolecules that contribute to the progression of HCC. A recent study found that LEF1-AS1 has a significantly higher expression in HCC tissues in comparison to adjacent normal tissues. The relationship between lncRNA LEF1-AS1 expression and clinicopathological parameters was analyzed, and the results showed that the expression of lncRNA LEF1-AS1 was significantly correlated with TNM staging, tumor size, and lymph node metastasis. Meanwhile, it had little correlation with age and gender (Dong et al., 2020).

### Colorectal Cancer

Increasing evidence suggests that lncRNAs participate in diverse cancers. Among these, LEF1-AS1 was recently recognized as an oncogenic lncRNA in CRC (Chen et al., 2017). Colorectal cancers are some of the most common cancers worldwide, with more than one million new cases diagnosed each year (Siegel et al., 2018). Among them, colon cancer is the third globally most common malignant tumor in men and the second most common malignant tumor in women, with nearly 1.2 million new cases and 600,000 deaths each year (Ku et al., 2012). The morbidity and mortality associated with CRC in China are increasing year by year. The high mortality rate is due to the late diagnosis and rapid metastasis (Siegel et al., 2017a). In order to improve the early diagnosis rate of CRC and reduce its high metastatic rate, it is crucial to screen new biomarkers that can predict the diagnosis and treatment of CRC, and improve its prognosis.

Dysregulated lncRNA spectrum and metastasis-related lncRNA were identified in colorectal cancer through genome-wide analysis, and it was found that LEF1-AS1 expression might be closely linked to the development of colorectal cancer. Successive studies were then performed on LEF1-AS1 and CRC. Shi et al. collected 91 CRC tissue samples and 60 plasma samples to evaluate the expression of LEF1-AS1. The results showed the upregulation of LEF1-AS1 expression. In addition, the expression of LEF1-AS1 in CRC tissues was significantly correlated with the expression of lymph node metastasis and Ki67. Plasma LEF1-AS1 expression was also associated with carcinoembryonic antigen (CEA) levels. Further studies found that the OS and DFS of CRC patients with higher expression levels of LEF1-AS1 were significantly shorter (Shi et al., 2019). Other studies have also supported these findings, and pointed out that high LEF1-AS1 levels are also significantly related to higher histological grades and Dukes stages (Cheng Y. et al., 2020; Sun et al., 2020).

### Oral Squamous Cell Carcinoma

OSCC, as one of the most common malignancies that originate in the oral mucosa, is the main component of oral cancer (Siegel et al., 2014; Chai et al., 2020), and presents a very high mortality rate and extremely low survival rate (Huang et al., 2020). Zhang et al. explored the connection between LEF1-AS1 and OSCC (Zhang C. et al., 2019). First, they evaluated the expression of LEF1-AS1 in OSCC tumor tissue and adjacent normal tissues. The results revealed that the expression of LEF1-AS1 was upregulated in OSCC tissues, and this was closely related to poor prognosis. In addition, the expression of LEF1-AS1 was found to be associated with tumor staging. These results suggest that LEF1-AS1 may be a prognostic marker of OSCC.

### Ovarian Cancer

One of the most common gynecological tumors causing death in women is ovarian cancer (Yokoi et al., 2018), which often has poor prognosis, and most women are diagnosed at an advanced stage (Menon et al., 2021). The role of lncRNAs in cancer development has received growing attention. Zhang and his team (Zhang and Ruan, 2020) found that LEF1-AS1 was closely related to OC, regulating its occurrence and development. They collected 62 pairs of OC tissues and adjacent non-tumor controls, and found increased levels of LEF1-AS1 in ovarian cancer tissues. In addition, LEF1-AS1 was highly expressed in ovarian cancer tissues with lymph node metastasis and advanced stage, and patients with high LEF1-AS1 expression had low overall survival.

### Esophageal Squamous Cell Carcinoma

Esophageal cancer is globally the eighth most common tumor and the sixth leading cause of tumor-related deaths (Siegel et al., 2017b). In China, the main histological type of esophageal cancer is esophageal squamous cell carcinoma (ESCC), which is a type of cancer with poor prognosis and a limited understanding of its molecular etiology (Li B. et al., 2021). Recently, lncRNA LEF1-AS1 has been shown to be dysfunctional in many cancer types. The evaluation of the level and function of LEF1-AS1 in ESCC showed that LEF1-AS1 upregulation was observed in 136 (73.5%) cases of ESCC specimens. LEF1-AS1 expression was associated with clinical stage (*p* = 0.008) and lymph nodes metastasis (*p* = 0.009), and higher LEF1-AS1 expressions were correlated with poor prognosis in ESCC patients. The results of univariate and multivariate analyses to further determine the prognostic impact of LEF1-AS1 expression indicated that LEF1-AS1 can be regarded as an independent poor prognostic factor of ESCC (Zong et al., 2019).

### Other Cancers

Certain studies reported a significant overexpression of LEF1-AS1 in osteosarcoma, prostate cancer, and retinoblastoma (Liu et al., 2019; Li W. et al., 2020; He and Qin, 2020; Lu et al., 2020). He et al. pointed out that high expression of LEF1-AS1 predicted poor prognosis in retinoblastoma. They found that LEF1-AS1 expression levels in retinoblastoma and IIRC D-E patients were remarkably increased compared with IIRC A-C patients. In addition, LEF1-AS1 has been reported to be downregulated in bone marrow-related disorders, while no specific clinical studies have been conducted (Congrains-Castillo et al., 2019; Szikszai et al., 2020).

## *In Vivo* Studies

Increasing evidences have demonstrated the effects of LEF1-AS1 silencing or over-expression in xenograft animal models. In lung cancer, the tumor size of sh-LEF1-AS1 was significantly smaller compared to the control group, and Ki-67 expression was also significantly reduced (Wang et al., 2019). Yang et al. evaluated the *in vivo* metastatic efficacy of LEF1-AS1 in a mouse model, and more metastatic nodules were detected in the mice injected with LEF1-AS1-overexpressed A549 cells group compared with those of the control group, implying that LEF1-AS1 could serve as an oncogenic driver in the pathogenesis of NSCLC (Yang et al., 2019). In colorectal cancer, researchers injected LEF1-AS1 into nude mice and then monitored the growth of tumor xenografts. They found that LEF1-AS1 may be beneficial to tumor growth and lung metastasis *in vivo* (Sun et al., 2020). Qi et al. also confirmed the tumorigenic effect of LEF1-AS1 in colorectal cancer (Qi et al., 2021). In terms of HCC, *in vivo* experiments on tumor-bearing nude mice exhibiting positive WNK1 expression confirmed the interference effect of lncRNA LEF1-AS1 on HCC (Dong et al., 2020). In addition, studies on OSCC (Zhang C. et al., 2019), prostatic carcinoma (Liu et al., 2019; Li W. et al., 2020), ESCC (Zong et al., 2019), glioma (Cheng Z. et al., 2020), and GBM (Wang et al., 2017) indicated that LEF1-AS1 can promote tumor formation *in vivo*.

## Cell Line Studies

LncRNAs can function as ceRNAs targeting specific microRNAs and then acting through complex molecular mechanisms. We have already discussed the level of LEF1-AS1 in different kinds of cancer and its clinical-pathological features above, and *in vivo* experiments have further verified the tumorigenic effect of LEF1-AS1. In the following sections, we aim to describe the role and related mechanisms of this lncRNA in different cancer cell lines (Supplementary Table S2).

### Glioma

Cheng et al. pointed out that the expression level of LEF1-AS1 was markedly elevated in cell lines, which is consistent with the results for glioma tissues. Besides, the knockdown of LEF1-AS1 could inhibit tumor cell proliferation while activating apoptosis in glioma cells *in vitro*. This was primarily achieved through the upregulation of HIGD1A expression by targeting miR-489-3p (Cheng Z. et al., 2020). In GBM, researchers also found that low LEF1-AS1 gene knockout significantly inhibited the growth state of GBM cells and reduced their malignancy (Wang et al., 2017).

### Non-Small-Cell Lung Cancer

LEF1-AS1 inhibits cell apoptosis and promotes NSCLC proliferation mainly through two pathways: the miR-221/PTEN axis and the miR-489/SOX4 axis. In NSCLC cells, researchers observed that the overexpression of LEF1-AS1 caused the downregulation of PTEN expression and the upregulation of miR-221 expression. Studies have revealed that miR-221 can directly target PTEN. Therefore, researchers considered that the carcinogenic effect of LEF1-AS1 is mainly realized through the miR-221/PTEN signaling pathway (Xiang et al., 2020), and LEF1-AS1 can also induce EMT progression through the miR-489/SOX4 axis (Yang et al., 2019).

### Hepatocellular Carcinoma

Dong et al. demonstrated through a series of cellular experiments that the overexpression of LEF1-AS1 affects the tumor state and is conducive to its proliferation, migration, and invasion. The results of a tube-forming assay showed that LEF1-AS1 also increases angiogenesis in the human umbilical vein endothelial cell (HUVEC). The specific mechanism of LEF1-AS1 promoting HCC may be through the miR-136-5p/WNK1 axis (Dong et al., 2020).

### Colorectal Cancer

**FIGURE 1:**
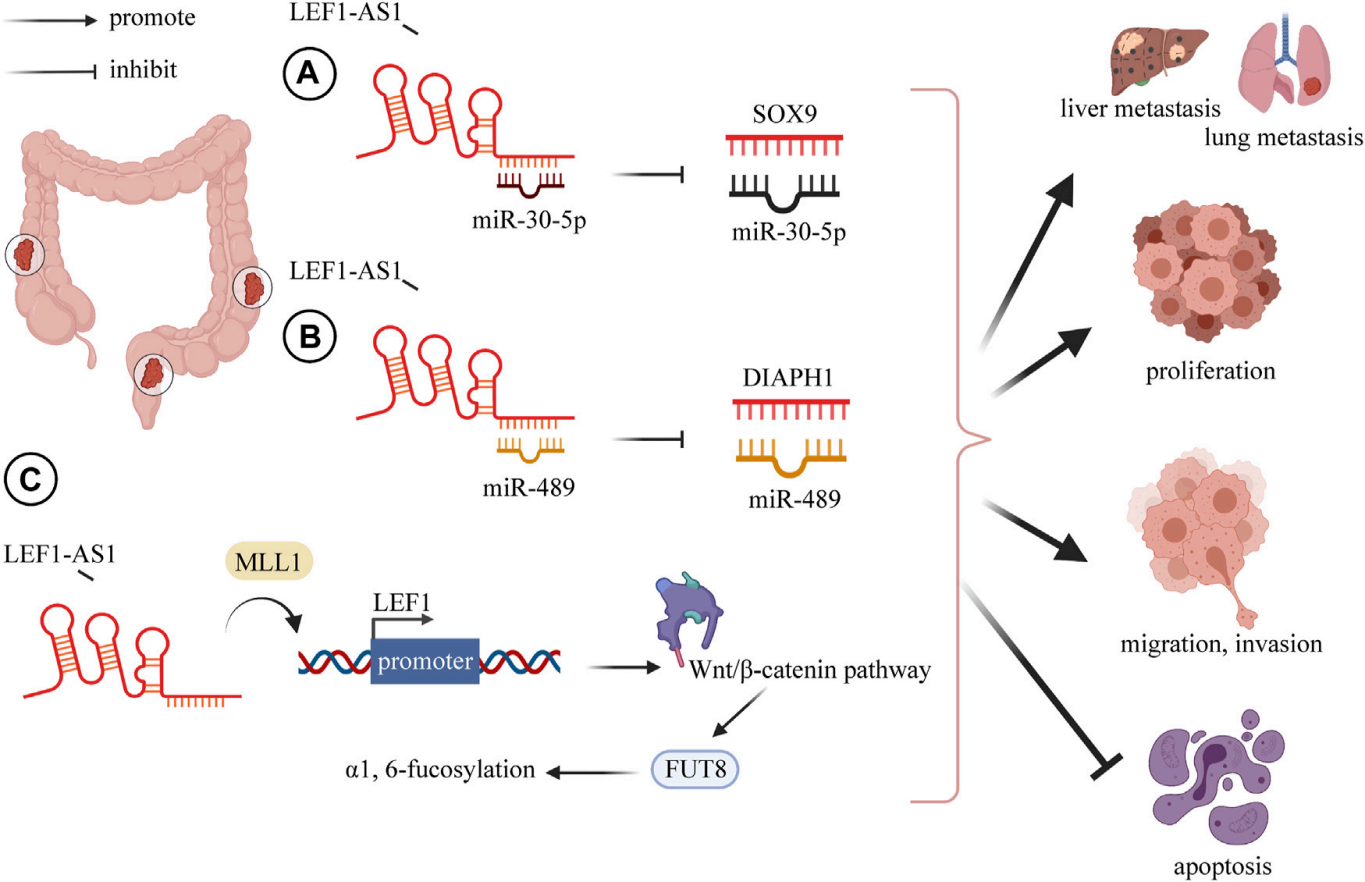
LncRNA LEF1-AS1 plays a carcinogenic role in CRC, overexpression of LEF1-AS1 promotes CRC proliferation, invasion, migration, distant metastasis (liver and lung), and inhibits apoptosis. **(A)** LEF1-AS1 binds to miR-30-5p and mediates the expression of SOX9 by negatively regulating miR-30-5p. **(B)** LEF1-AS1 binds to miR-489 and mediates the expression of DIAPH1 by negatively regulating miR-489. **(C)** LEF1-AS1/LEF1/FUT8 axis mediates colorectal cancer progression by regulating α1, 6-fucosylation via Wnt/β-catenin pathway. Abbreviations: LEF1-AS1, lymph enhancer-binding factor 1-antisense RNA 1; CRC, colorectal cancer; SOX4, SRY-related HMG box transcription factor 4; DIAPH1, diaphanous related formin 1.

Many studies have indicated that the lncRNA LEF1-AS1 has a carcinogenic role in the pathogenesis of colorectal cancer (Shi et al., 2019; Cheng Y. et al., 2020; Sun et al., 2020; Qi et al., 2021). Cheng et al. downregulated LEF1-AS1 through si-LEF1-AS1 and found that the growth, migration, invasion, and EMT of CRC cells were decreased, and apoptosis was elevated. They further explored the specific mechanism and established that LEF1-AS1 mainly acts through ceRNA on miR-489 to increase the DIAPH1 level (Cheng Y. et al., 2020). This is consistent with the findings of Qi et al. (2021). In colon cancer, a series of cell experiments was conducted to verify the function of LEF1-AS. The results showed that LEF1-AS1 negatively regulated miR-30-5p and SOX9 was the downstream target of miR-30-5p. Specifically, the overexpression of LEF1-AS1 increased SOX9 expression, and the recovery of SOX9 weakened the effects caused by LEF1-AS1 knockdown on cell migration, invasion, and non-anchoring growth (Sun et al., 2020) (Figure 1).

### Oral Squamous Cell Carcinoma

LEF1-AS1 was significantly elevated in the OSCC cell line. Functionally, the downregulation of LEF1-AS1 inhibits cell survival, proliferation, and migration, but enhances apoptosis. The cell cycle was also significantly affected. The downregulation of LEF1-AS1 significantly increased the G0/G1 phase arrest ratio, and the cells stayed in this period for longer (Zhang C. et al., 2019).

### Other Cancers

**FIGURE 2:**
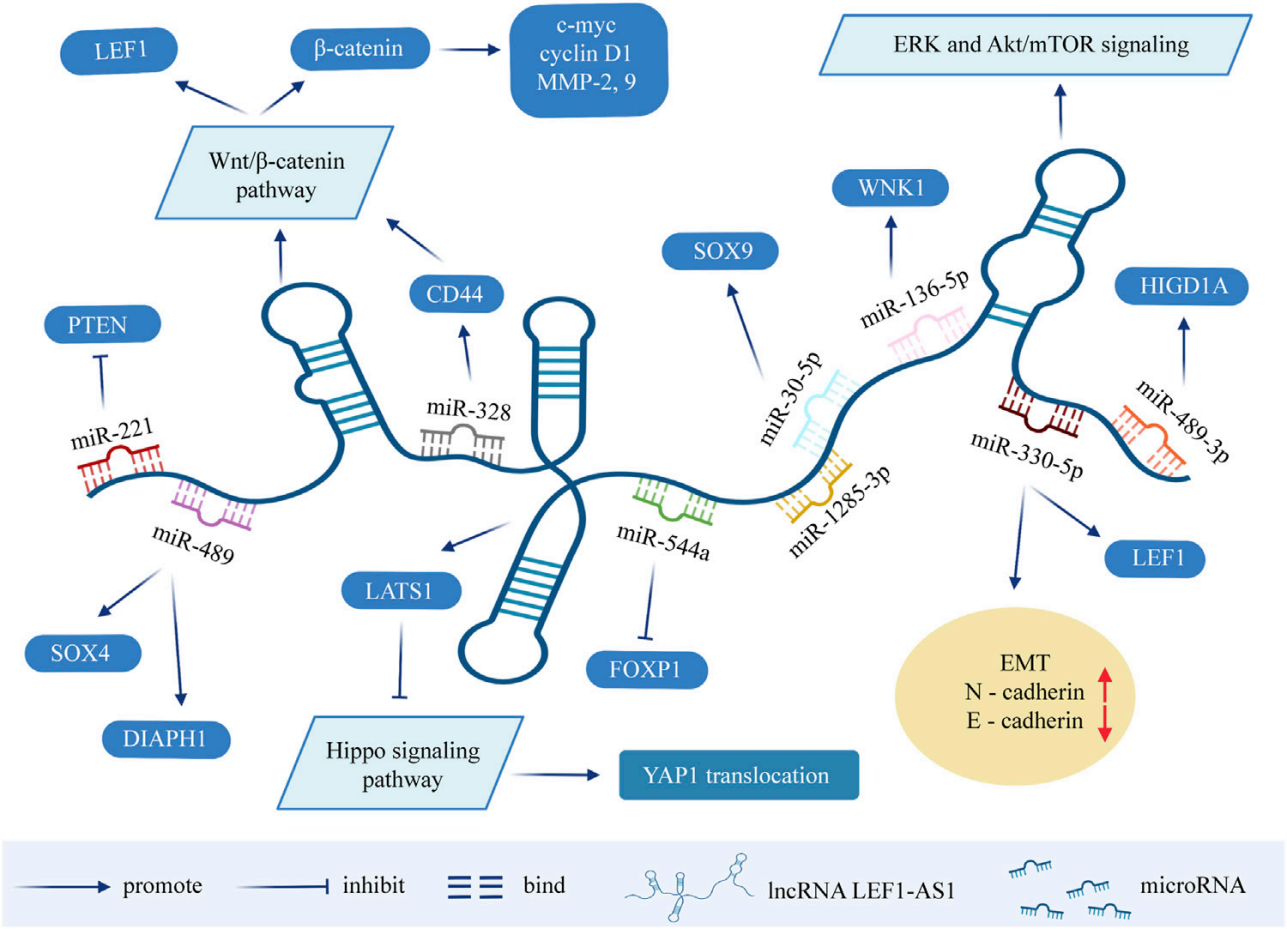
Molecular mechanisms and related signaling pathways of lncRNA LEF1-AS1 in cancers. MicroRNA can cause gene silencing by binding to mRNA, while ceRNA can regulate gene expression by competitively binding to microRNA. LEF1-AS1 can be used as ceRNA, sponge miRNA, thereby affecting the expression of target genes. LEF1-AS1 also participates in signaling pathways such as ERK and Akt/mTOR signaling pathway, Wnt/β-catenin pathway, and Hippo signaling pathway. Abbreviations: LEF1-AS1, lymph enhancer-binding factor 1-antisense RNA 1; ceRNA, competing endogenous RNA; EMT, epithelial-mesenchymal transition.

In lung cancer (Wang et al., 2019), osteosarcoma (Lu et al., 2020) and retinoblastoma (He and Qin, 2020), it has been reported that LEF1-AS1 is an upregulated oncogene promoting tumor progression. One study on ovarian cancer showed that the downregulation of LEF1-AS1 gene inhibited the proliferation, migration, and invasion of ovarian cancer cells. In terms of mechanism, LEF1-AS1 is the sponge of miR-1285-3p, which exerts its carcinogenic function by suppressing miRNA activity through interaction with miR-1285-3p (Zhang and Ruan, 2020). Moreover, Liu and his colleagues found that the silencing of LEF1-AS1 inhibits the occurrence and development of prostate cancer through blocking LEF1 as a molecular sponge for miR-330-5p (Liu et al., 2019). On the basis of previous studies, Li et al. further demonstrated that LEF1-AS1 promotes the angiogenesis of prostatic carcinoma (Li W. et al., 2020). Although LEF1-AS1 plays a carcinogenic role in most tumors, it has been found to be a cancer suppressor for bone marrow malignancies. It is significantly overexpressed in normal hematopoietic stem cells, but is barely detected in myeloid malignant cells. Cell experiments showed that LEF1-AS1 can inhibit the proliferation of bone marrow malignant tumors, and plays a protective role in the occurrence and development of tumors (Congrains-Castillo et al., 2019). The above findings could provide novel aspects for the diagnosis and targeted therapy of patients with cancer (Figure 2).

## Signaling Pathway Associated With LEF1-AS1

As mentioned above, LEF1-AS1 can be used as a ceRNA sponging miRNAs to downregulate the level of miRNAs, and thus affect the transcription and translation of target genes. Many target genes are widely involved in a variety of signaling pathways, such as the ERK (extracellular regulatory protein kinase) and Akt/mTOR signaling pathway, the Wnt/β-catenin pathway, and the Hippo signaling pathway, which are involved in the process of tumor occurrence and development. In the previous section, this review summarized the molecular mechanisms of LEF1-AS1 in exerting the biological functions in tumors (Supplementary Table S2). In the next section, we briefly introduce several signaling pathways involved in LEF1-AS1, so as to provide some ideas for finding tumor therapeutic targets.

### ERK and Akt/mTOR Signaling Pathway

The dysfunction of PI3K/AKT/mTOR is common in a variety of human malignancies, including renal cell carcinoma (Ke et al., 2017), lung cancer (Jian et al., 2019), breast cancer (Kanumuri et al., 2020), and HCC (Wu et al., 2018). It functions by targeting genes as a downstream molecule of the PI3K/AKT signal. AKT increases the activation of mTOR by inhibiting the phosphorylation of the mTOR inhibitor, which is the nodular sclerosis complex (TSC) protein 1/2 (Jin et al., 2019). ERK, an important member of the MAPK family, mainly includes ERK1 and ERK2, is activated by growth factors, and regulates cell proliferation and survival. Most ERK substrate molecules are proteins that regulate the cell cycle. Moreover, the inhibition of ERK activity can downregulate the expression of c-myc and promote apoptosis (Asati et al., 2016; Yue et al., 2019). In conclusion, the Akt/mTOR and ERK pathways play a fundamental role in cancer, and drugs targeting these pathways are expected to provide new options for tumor treatment. Wang et al. (Wang et al., 2017) found that the LEF1-AS1-mediated downregulation of tumor inhibition in GBM cells may be achieved by reducing ERK and Akt/mTOR signaling activities.

### Wnt/β-Catenin Pathway

This pathway inhibits the degradation of *β*-catenin that regulates the transcription of multiple genes, and many of the regulated genes are closely linked to cancer (Moon et al., 2002; Tanaka et al., 2002; Zhou and Liu, 2015). Li and his colleagues pointed out that LEF1-AS1 promotes the metastasis of prostatic carcinoma through the Wnt/β-catenin pathway (Li W. et al., 2020). Similarly, He et al. (He and Qin, 2020) found that silent LEF1-AS1 inhibited the biological function of retinoblastoma cells by inhibiting the expressions of *β*-catenin and LEF1. The Wnt/β-catenin pathway has also been confirmed to be involved in the occurrence and progression of osteosarcoma (Lu et al., 2020) and colorectal cancer (Qi et al., 2021).

### Hippo Signaling Pathway

The pervasively activated Hippo pathway has been recognized to have essential roles in different cancer types (Goyal et al., 2021; Pham et al., 2021), and is involved in cancer initiation and progression, as well as resistance to cancer treatment (Liu Q. et al., 2021). There are many components in the mammalian Hippo pathway, including the kinase cascade, transcriptional co-activation factors YAP, MST1/2 and LATS1/2, and downstream effector (Hong et al., 2016). These core components of the Hippo pathway control the transcriptional processes involved in different functions, such as proliferation, mobility, and differentiation (Ma et al., 2019). In a study on OSCC (Zhang C. et al., 2019), researchers found that LEF1-AS1 directly interacts with LATS1 and affects downstream MOB and YAP1 to inactivate the Hippo signal. It was demonstrated that LEF1-AS1 has a carcinogenic effect in OSCC by inhibiting the Hippo signaling pathway.

## Conclusion

LncRNAs are involved in the regulation of various biological functions in the nucleus and cytoplasm, involving developmental processes in the fields of immunology, neurobiology, cancer, and stress, and are also key regulators of cell proliferation and apoptosis. Researches on the role of lncRNAs in cancer are increasingly popular. The occurrence and development of cancer can be mediated by various mechanisms involving lncRNAs, which is mainly through epigenetic regulation, the activation of carcinogenic or tumor suppressor pathways, and so on.

This review mainly discusses the role and research progress of lncRNA LEF1-AS1 in a variety of human tumors. According to the literature, LEF1-AS1 is highly expressed in most tumors, and its expression is reduced in bone marrow malignant tumors. The high expression of LEF1-AS1 is associated with the low survival rate of patients with cancers such as glioma, osteosarcoma, lung cancer, ovarian cancer, colorectal cancer, esophageal squamous cell carcinoma, etc., suggesting that LEF1-AS1 can be used as a biomarker for early detection and prognostic evaluation. In addition, a large number of cell experiments further verified that LEF1-AS1 is involved in cell proliferation, migration, invasion, apoptosis, angiogenesis, and the process of the epithelial cell to mesenchymal transformation. Gao et al. also found that LEF1-AS1, as a miR-10a-5p regulatory factor, enhances the expression of MSI1 in liver cancer cells by activating the AKT signaling pathway, and promotes resistance to chemotherapy. In short, with LEF1-AS1 as a starting point, a series of related applications can be developed with respect to tumor detection, diagnosis, treatment, and prognosis. However, the current research on LEF1-AS1 has certain defects. First of all, there is still a lack of research on LEF1-AS1 in breast cancer, pancreatic cancer, and other tumors. There are very few studies in hematological malignancies. Secondly, more research is still being carried out *in vitro*, lacking clinical correlation. In short, it is worth more in-depth research in the future.
