# Supplementary material for: Current Research Progress of the Role of LncRNA LEF1-AS1 in a Variety of Tumors
Source: Front Cell Dev Biol. 2021 Dec 20;9:750084. doi: 10.3389/fcell.2021.750084 (PMC8721001; doi:10.3389/fcell.2021.750084)
Supplement: Supplementary file 1 [file DataSheet2.docx]

**Table 2 The functional characterization and molecular mechanisms of LEF1-AS1 in a variety of cancers**

| **Cancer type** | **Assessed cell lines** | **Functional role(validated)** | **Related genes/pathways** | **Property** | **Refs** |
| --- | --- | --- | --- | --- | --- |
| Glioblastoma (GBM) | 293FT, U251, U87 | Proliferation, invasion, and anti-apoptosis | ERK and Akt/mTOR signaling | Oncogene | Wang et al., 2017 |
| Non-Small﻿-Cell Lung Cancer (NSCLC) | NCI-H1993 (H1993, LUAD), NCI-H1581 (H2170, LUSC) | Proliferation, anti-apoptosis | miR-221/PTEN axis | Oncogene | Xiang et al., 2020 |
|  | H1299, A549 | Proliferation, migration, invasion, metastasis, and anti-apoptosis | miR-489/SOX4 axis | Oncogene | Yang et al., 2019 |
| Lung cancer | BEAS-2B, H1299, A549, H1975, SPC-A-1 | Proliferation, migration, and invasion | miR-544a/FOXP1 axis | Oncogene | Wang et al., 2019 |
| Hepatocellular carcinoma (HCC) | HepG2, Hep3B, Huh7, SK‐HEP‐1, THLE‐3 | Proliferation, migration, invasion, and HUVEC angiogenesis | miR‐136‐5p/WNK1 axis | Oncogene | Dong et al., 2020 |
| Osteosarcoma | 143B, MG63, U‐2OS, OS‐9901, NHOst | Proliferation, migration, and invasion | Wnt signaling pathway, HNRNPL | Oncogene | Lu et al., 2020 |
| Myeloid malignancy | HL60, Hela | Inhibited proliferation | / | Suppressor | Congrains-Castillo et al., 2019 |
| Colon cancer | COLO320, SW480, SW1417, SW948, T84, HT29, HEK293T | Migration, invasion, and metastasis | miR-30-5p/SOX9 axis | Oncogene | Sun et al., 2020 |
| Colorectal cancer (CRC) | SW1116, HCT116, LOVO, SW480, FHC | Proliferation, migration, invasion, metastasis, and anti-apoptosis | miR-489/DIAPH1 axis | Oncogene | Cheng et al., 2020a |
|  | HCT-8, Caco2, SW480, SW620, LOVO, FHC | Proliferation, migration, invasion, and metastasis | LEF1-AS1/LEF1/FUT8 axis, Wnt/β-catenin pathway | Oncogene | Qi et al., 2021 |
| Oral squamous cell carcinoma (OSCC) | NHOK, SCC9, FADU, SCC25, SCC1, TU183, HSU3, OEC-M1, SNU1041, SCC4, SCC15 | Proliferation, migration, cell cycle, and anti-apoptosis | LEF1-AS1/LATS1/YAP1 axis, Hippo signaling pathway | Oncogene | Zhang et al., 2019a |
| Ovarian cancer (OC) | OVCAR5, A2780, SKOV3, OVCAR3, IOSE80 | Proliferation, migration, invasion, and metastasis | miR-1285-3p | Oncogene | Zhang and Ruan, 2020 |
| Prostatic carcinoma | PC3, DU145, RWPE | Proliferation, migration, invasion, metastasis, and angiogenesis | Wnt/β-catenin pathway, miR‑328/CD44 axis | Oncogene | Li et al., 2020b |
|  | PC3, 22RV1, RWPE‐1 | Proliferation, migration, invasion, metastasis, and EMT | miR‐330‐5p/LEF1 axis | Oncogene | Liu et al., 2019 |
| Glioma | U251, SWO38, U373MG, HEB, NHA | Proliferation, and anti-apoptosis | miR-489-3p/HIGD1A axis | Oncogene | Cheng et al., 2020b |
| Retinoblastoma | ARPE-19, SO-Rb50, HXO-RB44 | Proliferation, migration, and invasion | Wnt/β-catenin pathway | Oncogene | He and Qin, 2020 |

The main experimental techniques used in functional verification are as follows: MTT assay, CCK-8, Colony formation assay, Cell cycle analysis, Scratch test, Transwell assays, Flow cytometry, *In vitro* tube formation assays, *In vivo* xenograft experiments, Western blot, RT-qPCR.
