# Supplementary material for: Current Research Progress of the Role of LncRNA LEF1-AS1 in a Variety of Tumors
Source: Front Cell Dev Biol. 2021 Dec 20;9:750084. doi: 10.3389/fcell.2021.750084 (PMC8721001; doi:10.3389/fcell.2021.750084)
Supplement: Supplementary file 2 [file DataSheet1.docx]

**Table 1 The expression of LEF1-AS1 and its clinical significance in different cancers.**

| **Cancer type** | **Number of cases** | **Expression (Tumor vs. Normal)** | **Clinical significance** | **Overexpression of LEF1-AS1 associated with prognosis** | **Refs** |
| --- | --- | --- | --- | --- | --- |
| Glioblastoma (GBM) | 10 GBM tissues and 3 normal brain tissues | Up | / | Poor | Wang et al., 2017 |
| Non-Small﻿-Cell Lung Cancer (NSCLC) | 62 cases of NSCLC patients included 34 cases of LUAD and 28 cases of LUSC. | Up | / | / | Xiang et al., 2020 |
|  | 48 paired NSCLC tissues and adjacent non-tumor tissues | Up | Tumor size, and TNM stage | Poor | Yang et al., 2019 |
| Lung cancer | 48 pairs of lung cancer tissues and adjacent normal tissues | Up | / | Poor | Wang et al., 2019 |
| Hepatocellular carcinoma (HCC) | 121 pairs of HCC tissues and adjacent normal tissues | Up | Tumor size, TNM stage, and lymph node metastasis | / | Dong et al., 2020 |
| Osteosarcoma | 40 cases of osteosarcoma patients | Up | / | Poor | Lu et al., 2020 |
| Myeloid malignancy | / | Down | / | / | Congrains-Castillo et al., 2019 |
| Colon cancer | 50 pairs of colon cancer samples and matched tumor-adjacent tissues | Up | Dukes staging, and lymph node metastasis | Poor | Sun et al., 2020 |
| Colorectal cancer (CRC) | 116 CRC tumor specimens and matched adjacent normal tissues | Up | Histological grade, and lymph nodes metastasis | Poor | Cheng et al., 2020a |
|  | plasma was collected from 60 CRC patients and 34 matched healthy volunteers, 91 paired CRC tissues and adjacent non-tumor tissues | Up | Lymph node metastasis, Ki67 expression, and CEA levels | Poor | Shi et al., 2019 |
|  | 36 paired CRC tissues and adjacent non-tumor tissues | Up | / | / | Qi et al., 2021 |
| Oral squamous cell carcinoma (OSCC) | 88 pairs of OSCC tumor tissues and adjacent non-tumor tissues | Up | Stage | Poor | Zhang et al., 2019a |
| Ovarian cancer (OC) | 62 pairs of OC tissues and adjacent normal tissues | Up | Lymph node metastasis, and advanced stages | Poor | Zhang and Ruan, 2020 |
| Prostatic carcinoma | 45 cases of AIPC samples | Up | / | / | Li et al., 2020b |
|  | 58 cases of radical resection of Pca and their para‐cancerous tissues | Up | / | / | Liu et al., 2019 |
| Glioma | 46 pairs of glioma tissues and adjacent normal tissues | Up | / | Poor | Cheng et al., 2020b |
| Esophageal squamous cell carcinoma (ESCC) | 185 pairs of ESCC tissues and adjacent normal tissues | Up | Lymph node metastasis, and clinical stage | Poor | Zong et al., 2019 |
| Retinoblastoma | 42 pairs of retinoblastoma tissues and adjacent normal tissues | Up | / | Poor | He and Qin, 2020 |
